# DDA-bench: a manually curated database for benchmarking datasets and baseline performance values in predicting drug-disease associations

**DOI:** 10.3389/fgene.2025.1755600

**Published:** 2026-01-07

**Authors:** Han Xing, Yongjian Zhao

**Affiliations:** School of Airspace Science and Engineering, Shandong University, Weihai, Shandong, China

**Keywords:** baseline performance, benchmarking dataset, drug repurposing, drug-disease association, performance evaluation

## Abstract

Predicting drug-diseases associations provides hints in developing new drugs. Various computational methods have been developed. To develop better models for predicting drug-disease associations, two types of key resources must be obtained, the benchmarking dataset and the baseline performances. Collecting these resources usually requires extensive labors in reading literatures and extracting relevant information from the literature manually. We developed DDA-Bench databases services, which curates commonly used benchmarking datasets and up-to-date performance values from baseline studies. We analyzed data records in DDA-Bench database. We proposed that performance variations for a given method in the context of different reports should be noticed. The impact of dataset density on predictive performance exists, and should be considered in future studies. In addition, we release the DDA-Bench database to the public. The DDA-Bench database saves time and efforts in constructing data basis for developing new models for predicting drug-disease associations. The DDA-Bench database can be accessed at https://dda.csbios.net.

## 1 Introduction

Over the last few years, human civilization was impacted by several pandemics. Pharmaceutical industry contributed a lot in fighting these pandemics by providing vaccines, drugs, and other therapeutical treatments (Witek, 2021). Traditional pharmaceutical drug development is incredibly expensive and time-consuming (Mohan et al., 2024). Clinical trials usually produce negative results, turning billions of dollars and tens of years’ time to nothing.

However, in some cases, the molecules in late clinical trials or already on the market, does not work for its designed indication, but work well for correcting other disorders (Begley et al., 2021). The well-known example is the Viagra, which was originally designed to treat chest pain, but eventually a solution of erectile dysfunction (Saranraj and Kiran, 2025). Minoxidil, which was originally designed to treat hypertension, becomes a treatment of hair loss, because of its obvious and common side effects (Suchonwanit et al., 2019). Thalidomide, which was withdrawn because it causes birth defects as a treatment for sickness in pregnant women, finally becomes an anti-cancer treatment against myeloma (Kim and Scialli, 2011). These examples implied that an approved molecules may affect human physiological system in various ways. Therefore, an approved molecule may potentially become treatments of other diseases, which was not its original purpose. This is called drug repurposing (Hussain et al., 2025). It is a much more efficient way of developing new drugs, because the repurposed molecule is already in late clinical trial stage or already on the market (Corsello et al., 2017).

Experiments that search for new indications of an approved molecule still requires tremendous amount of labor. Computational drug repurposing can provide the primary hints in the exploratory stage efficiently, which saves time and investment. Modern artificial intelligence technology works as a powerful accelerator in this process. A number of antiviral drugs, such as Paxlovid (Graham, 2021), Veklury (Zhou et al., 2020), and Lageviro (Mohanty et al., 2020), were developed and marketed within two or 3 years. This is unthinkable without the help of computational drug repurposing.

Computational drug-repurposing has become an overwhelming research field in bioinformatics, computational biology and computational chemistry. Hundreds of models were developed to find potential new drug indications from various heterogenous information about drugs, diseases, and other biological and physiological information. For example, PREDICT (Gottlieb et al., 2011), which is the pioneer study on predicting novel drug indications, formalized large-scale indication prediction by integrating drug-drug, disease-disease, and drug-disease similarity features to predict novel indications for approved molecules. Wu et al. proposed a holistic approach by integrating known drug-disease associations together with many other information (Wu et al., 2017). RWHNDR (Luo et al., 2019) prioritize candidate drugs for diseases, by using random walks to capture global features of drug-gene-disease heterogenous network. DisDrugPred (Xuan et al., 2019) utilized a non-negative matrix factorization algorithm to better predict drug-related candidate disease indications. DRIMC (Zhang et al., 2020) incorporates the Bayesian inductive matrix completion method in a latent space to predict drug-disease associations with higher accuracy. HGIMC (Yang M. et al., 2021) integrated bounded matrix completion to compensate the sparsity of heterogenous drug-disease association network.

Recent advances of deep representation learning, particularly deep graph neural networks, elevated these models to the next level. For example, deepDR (Jiang and Li, 2024) is an influential network-based model that integrates various relation types with multimodal autoencoders to learn DAHNGC drug embeddings and suggest repositioning candidates. REDDA (Gu et al., 2022), which enhances the drug-disease association representations by assembling three different attention mechanism on graph models, provided novel indication predictions that can be supported by literatures. DAHNGC (Zhong et al., 2023), which is based on graph convolutional neural networks, achieved better predictive performance in evaluations. HEDDI-Net (Su et al., 2025), which is a very recent graph-embedding architecture specifically designed for predicting drug-disease associations, outperforms many baseline predictors.

With all these advancement in computational models, it is an obvious challenge for a practical study to choose the best model. Here, the best may not be a unique answer in all scenarios, as different predicting models may be suitable for different target diseases. It is impossible to know which is the best, before trying all of these models. Even if we do not consider this kind of complex practical cases, simply comparing predictive performances of different models is already a challenging task.

Since all published works reported performance comparisons against, so-called, state-of-the-arts, methods, inconsistent performance values of the same method often appear in different reports. This may due to variations in evaluation processes, including variations on benchmarking datasets, differences in validation protocols, and differences in performance measure definitions. In particular, variations on performance values may naturally exist, because of the intrinsic stochastic variation of modern deep neural network models. Moreover, the random partition scheme in the commonly applied n-fold cross-validation protocols may also introduce performance variations, which makes the accurate reproduction of performance value impossible. Specifically, some works do not provide source code and dataset for reproducing purpose due to copyright, commercial, or policy restrictions. Readers usually cannot obtain original resources to reproduce the results in these works. In these cases, readers tend to reproduce results by their own program, not the original codes and data. This inevitably creates inescapable diversity in performance values. All these variations in performance values, which ought to be identical, brings an ever-growing chaos to developing new models for drug repurposing. This also makes the application of computational models in practical drug repurposing difficult, if not impossible.

Several studies make their benchmarking datasets open to the public. For example, the SCMFDD (Zhang et al., 2018) study provides the most commonly used benchmarking dataset, the B-Dataset (DDA18-269-598) and the L-Dataset (DDA18-1323-2834). Both datasets are assembled based on the CTD database (Davis et al., 2023). The B-dataset contains only highly connected nodes in the drug-disease association network, while the L-dataset with no restriction. The DrugNet (Martínez et al., 2015) dataset (DDA15-1490-4517) is a very comprehensive collection that is assembled from the DrugBank database (Law et al., 2014). It also called the D-Dataset or the DN-Dataset. It is the basis of another widely used benchmarking dataset, which is usually called the C-Dataset (Luo et al., 2016). The C-Dataset (DDA16-663-409) is built upon the D-Dataset and the F-Dataset. The F-Dataset (DDA11-593-313) was the first publicly available benchmarking dataset for predicting drug-disease associations (Gottlieb et al., 2011). Although these are only a part of the publicly available datasets, relationships between them are already complicated, not to mention that some datasets have different alias names in different studies. It is necessary to establish a common ground for developing and evaluating future models for predicting drug-disease associations.

In order to create fair and objective comparisons between a newly developed model and existing state-of-the art models, recent studies tend to apply three to four benchmarking datasets to evaluate their effectiveness. However, finding proper benchmarking datasets and baseline models is quite difficult. Although some methods claimed that they have datasets and codes available publicly, the links to these resources had been buried by the fast update of modern internet infrastructures. Therefore, missing comparisons on specific benchmarking datasets or against specific baseline models is becoming a common practice, due to the inability to retrieve a more comprehensive set of related resources. These missing comparisons may lead to biased results and conclusions.

To this end, we developed the DDA-Bench database, which is a manually curated collection of datasets and baseline performance values for predicting drug-disease associations. DDA-Bench is designed to be a one-stop solution for scheduling the evaluation and comparison in developing models for predicting drug-disease associations. We included over a dozen benchmarking datasets that can be found and retrieved publicly. These datasets are kept on our server, as well as on Zenodo, for downloading publicly. All datasets are provided with the original link. We also included performance values from various studies in this database. These values are extracted and curated with the help of AI-based assistants. All values are manually reviewed before depositing in the DDA-Bench database.

## 2 Results and discussions

### 2.1 The DDA-bench database

We established the DDA-Bench database (https://dda.csbios.net). The DDA-Bench database is a collection of benchmarking datasets for predicting drug-disease associations, and baseline performance values that are reported in various conditions. We included a total of 13 benchmarking datasets in the DDA-bench database, along with 1180 records of performance values from 12 representative baseline studies. A total of 106,401 records of drug-disease associations are accumulated in the DDA-Bench database. This collection is sufficient for all comparison and evaluation purpose in developing new models for predicting drug-disease associations. A comprehensive list of the dataset names, source references, dataset sizes can be found in Supplementary Table S1.

**FIGURE 1:**
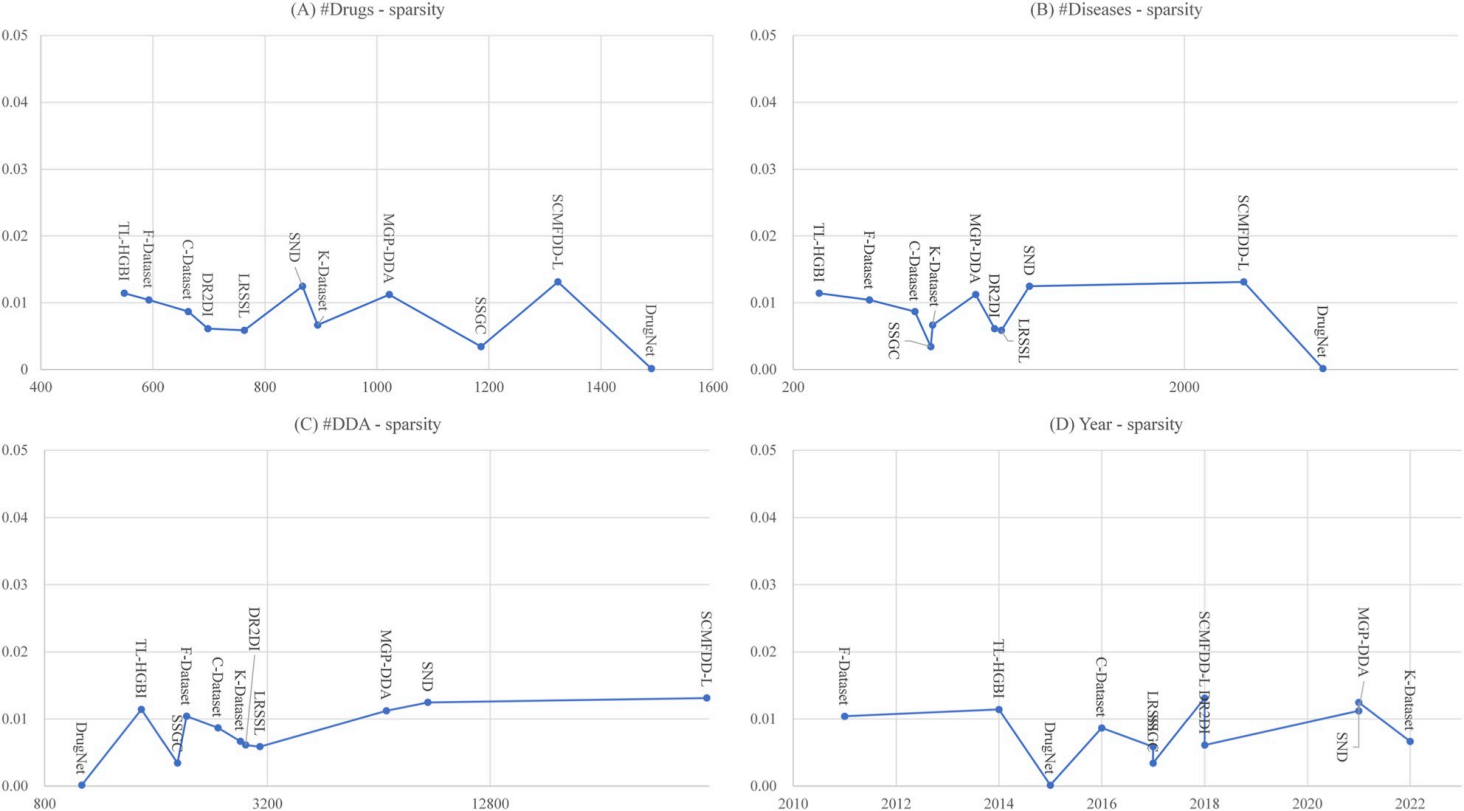
Dataset scale against dataset density. **(A)** The relationship between the number of drugs and density; **(B)** The relationship between the number of diseases and density; **(C)** The relationship between the number of DDA and density; **(D)** The relationship between the dataset releasing year and density. In each panel, the vertical axis is the density, while horizontal axis the dataset scale parameter.

We provided a parameter density to describe the proportion of known drug-disease associations in all drug-disease pairs. Figures 1A–C illustrated the relationship between the density and the number of drugs, number of diseases and number of known drug-disease associations. We can see that, although the number of drugs, the number of diseases and the number of drug-disease associations in different datasets varies in a large range, the density values of different datasets do not change much. This observation indicates that the number of known drug-disease associations in the public database increased in a steady rate along with the increment of the number of drugs. We also performed an additional analysis on the relationship between the releasing year and the density value of datasets Figure 1D. This analysis further confirmed our observation in Figures 1A–C. Even with the accumulation of records in the primary databases, such as DrugBank and CTD, the density of benchmarking datasets does not change much. The known drug-disease association occupy a very steady portion of all drug-disease pairs.

For future works, it would be useful to construct a universal benchmarking dataset that summarizes the common records from different datasets in the DDA-Bench database. However, since nearly all datasets have their own systems of drug and disease identifiers and their own data format, even with the help of current artificial intelligence, it requires very hard manual work to ensure the quality of such universal dataset. It is also nearly impossible to keep such kind of universal dataset updated in a long time. We hope the development of advanced AI assistant may finally finish this kind of work automatically, and update the dataset regularly.

### 2.2 The relationships among different datasets

**FIGURE 2:**
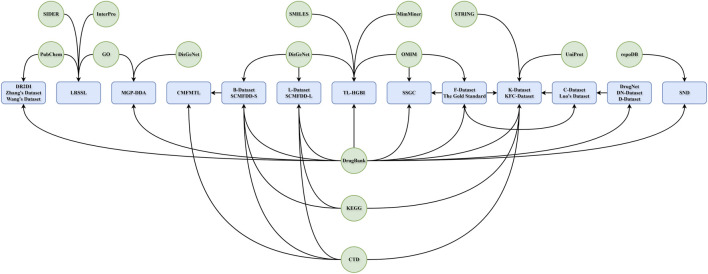
The information flowchart between benchmarking datasets and primary databases. The circles represent primary databases. The round corner rectangles represent benchmarking datasets. The arrow indicates information flow directions. Some benchmarking datasets are built upon other earlier benchmarking dataset. Most datasets obtain information from DrugBank, CTD and KEGG databases. Annotations are from various sources, due to the requirement of different reports.

We illustrated the relationships among different datasets, and their primary information source databases (Figure 2). All popular benchmarking datasets are curated from primary or secondary level databases. The most common source of information is the DrugBank, followed by the CTD database. KEGG, MeSH, PubChem, OMIM and GO databases are also used by multiple datasets as their primary information source. Since some datasets included multi-omics information in its disease/drug representations, information source provides not only drug-disease associations, but also related genomic and chemical information.

It should be noted that some datasets are derived from other datasets. The C-Dataset, the K-Dataset (DDA22-894-454), and SSGC (Wu et al., 2017) dataset (DDA17-1186-449) are all derived partially from the F-Dataset. Since F-Dataset is the earliest benchmarking dataset in public, it is natural that follow-up studies use this dataset as a foundation of their work. In addition, the C-Dataset has its information derived not only from the F-Dataset, but also from the D-Dataset. The D-Dataset is mainly based on the DrugBank database. The K-Dataset included information that is specific of C-Dataset. It also has records imported directly from the DrugBank database, making it a very comprehensive and very up-to-date benchmarking datasets. These relationships among different datasets are useful in choosing the working basis when developing new models for predicting drug-disease associations. The new models in these days, should be evaluated, at least, using the F-Dataset, the K-Dataset, and the B-Dataset. This combination covers the most core records (F-Dataset), the DrugBank up-to-date information (K-Dataset) and the CTD information (B-Dataset). Since the DrugBank and the CTD databases cover most of recorded drug-disease association in different modes, benchmarking a new model with these records will be necessary to have a big picture of its performance. The F-Dataset should be considered, as it is the most commonly applied dataset, which forms a common basis for performance comparison.

The names of these datasets are quite difficult to remember. Over years, many aliases were created for these datasets in different literatures. Although forerunners may be able to remember these names, and their actual meaning, including the scale and the source of the dataset, it is quite a difficult task for a student or a new scientist to remember these names, and aliases. Therefore, we propose that a systematic naming convention should be carried out in future studies. We add these names as the Dataset Standard Name in our DDA-Bench database. The standard name of a dataset is prefixed by DDA, with three numbers afterwards. These three numbers represent the year of its publication, the number of drugs and the number of diseases. We understand that this does not make the name easier to remember. But this name makes it easy to differentiate them, and to stop the chaos in dataset alias names. We provide the map of the standard name, the common name and common aliases of all datasets in DDA-Bench in Table 1. We add the standard name after mentioning each dataset for the first time in this paper.

**TABLE 1:** Dataset naming conventions.

Standard name	Common name	Aliases
DDA18-269-598	B-dataset	SCMFDD; SCMFDD-S
DDA18-1323-2834	L-dataset	SCMFDD; SCMFDD-L; L-dataset
DDA16-663-409	C-dataset	Luo’s dataset; MBiRW dataset
DDA22-894-454	K-dataset	KFC-dataset
DDA11-593-313	F-dataset	The gold standard; G-dataset
DDA17-763-681	LRSSL	N.A.
DDA21-1022-585	MGP-DDA	N.A.
DDA14-549-233	TL-HGBI	N.A.
DDA15-1490-4517	D-dataset	DrugNet; DN-dataset
DDA18-698-654	DR2DI	Zhang’s dataset; Wang’s dataset
DDA17-1186-449	SSGC	N.A.
DDA21-867-803	SND	N.A.
DDA20-269-598	CMFMTL	N.A.

### 2.3 Performance variation analysis

The prediction performances vary in different reports. Even for the same method on the same dataset, the performance values are different in different comparisons. This may be due to the different validation protocols and may also be due the randomness that is brought by the partitioning scheme in cross-validations. By collecting performance values from various literatures, our DDA-Bench database enabled us to see these variations quantitatively.

**FIGURE 3:**
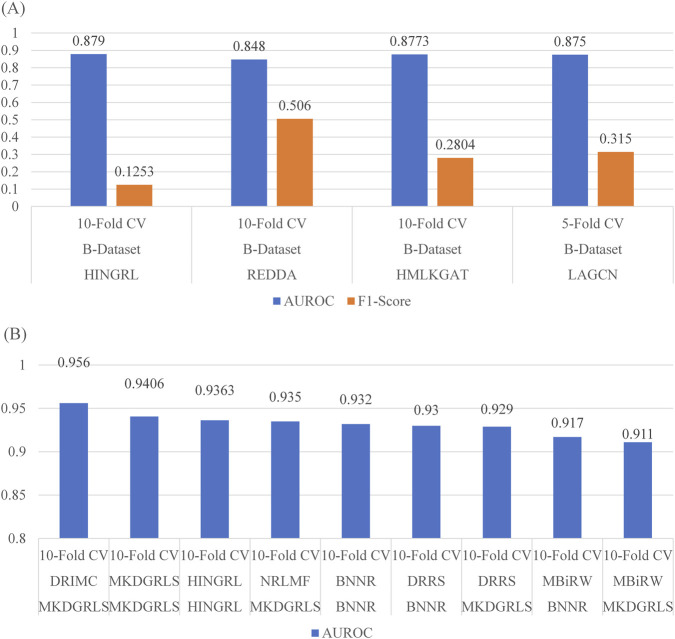
Performance variation analysis in different reports. **(A)** The performance of LAGCN in different reports. **(B)** The average performance on the F-Dataset from different reports. **(B)** In the first row of method name is the evaluated method. The second row of method name is the reporter.

We focus on the AUROC performance measure mainly in this analysis, as this is the best commonly reported performance measure that can be compared without the influence of the cut-off value across various literatures. Another measure we included is the F1-Score, which is regarded as a score that measures the performance of a classifier comprehensively. We first focus on the performance of a commonly used baseline method, LAGCN (Yu et al., 2021). The LAGCN method was used as the baseline performance in many follow-up studies. We choose the HINGRL (Zhao et al., 2022), REDDA, HMLKGAT (Li et al., 2024) and LAGCN in this comparison. The AUROC and the F1-Score were presented in Figure 3A. All methods in comparison reported the LAGCN performance on the B-Dataset. Three methods reported the 10-fold cross-validation results, while LAGCN report its own performance using 5-fold cross-validation. The variation of AUROC in different studies is minimal. The highest AUROC of LAGCN was reported by HINGRL, as 0.879, which is only 0.031 higher than the lowest value in the REDDA study. However, it is quite interesting to see that REDDA reported the highest F1-Score for LAGCN, which is significantly higher than all other three studies. It also should be noted that LAGCN does not report its own performance as the highest or the lowest in this comparison. Therefore, we believe LAGCN study objectively reported its own performance. Variations among different studies may only come from randomness in the partitioning scheme of a cross-validation. This is also why LAGCN is used as a common baseline.

Besides the choice of baseline method, telling which method is the state-of-the-art method on a given dataset is also a task that requires a lot of readings. Our DDA-Bench saves time in this process. We take the F-Dataset as an example. Our records show that the best reported AUROC on the F-Dataset was achieved by the DRIMC (Zhang et al., 2020) method. The value was reported by the MKDGRLS (Yang H. et al., 2021). This is followed by MKDGRLS reporting its own performance. The next one is the HINGRL reporting its own performance. The fourth and the fifth one are the BNNR (Yang et al., 2019) and DRRS (Luo et al., 2016) performances in the BNNR reports (Figure 3B). Therefore, when choosing state-of-the-art methods for comparisons on the F-Dataset, a best combination would be DRIMC, MKDGRLS, HINGRL, BNNR, and DRRS.

### 2.4 Meta-comparison on performances

Different studies report performance of a same method using different validation protocols, with different partition scheme. It is quite difficult to perform an objective evaluation that is with respect to every study. Our DDA-Bench provides an opportunity to carry out this kind of performance comparison. We call this a meta-performance comparison.

**FIGURE 4:**
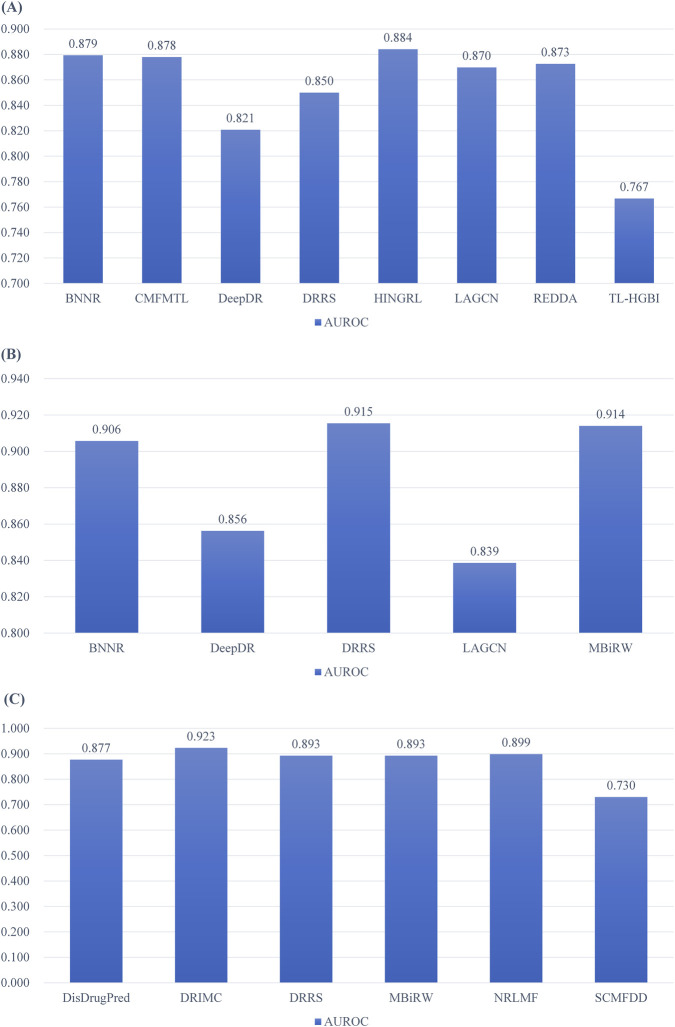
Meta performance comparisons. **(A)** Meta performance comparisons on B-Dataset. **(B)** Meta performance comparisons on F-Dataset. **(C)** Meta performance comparisons on C-Dataset. Values in this figure is the average performance of a given method on a given dataset in different reports.

We take the B-Dataset as an example. We collect studies that reports AUROC values on this given dataset. For each method in comparison, we average its performance in different studies other than the method of its own report. This is to keep the result objective. The meta-comparison of performances on the B-Dataset is in Figure 4A.

We now see that on the B-Dataset, if we take the performance variation of a method in different reports into consideration. The AUROC values of BNNR, CMFMTL (Huang et al., 2020), HINGRL, LAGCN, and REDDA are quite similar. The AUROC values of DeepDR, DRRS and TL-HGBI (Wang et al., 2014) is lower than the state-of-the-art level. In particular, when developing new models based on the B-dataset, the performance of HINGRL should be marked as a target.

We can also take the F-Dataset as another example (Figure 4B). In the meta-comparison, the BNNR, DRRS, and MBiRW (Luo et al., 2016) achieved over 0.9 in terms of AUROC. While other methods like DeepDR and LAGCN cannot reach a similar level. Therefore, on the F-Dataset, the state-of-the-arts methods should reach at least 0.9 in terms of AUROC. For the C-Dataset, the meta-comparison shows that all state-of-the-arts methods reached around 0.9 in terms of AUROC, except for SCMFDD (Figure 4C).

The meta-comparison results clearly indicate which methods are the state-of-the-art methods on a given benchmarking dataset. Specifically, since the meta-comparison eliminated the self-reported performance of every method, it is more objective than using only the originally reported values. In addition, this meta-performance can be regarded as a long-term test for a given method incorporating the factor of randomization, which provides more stable results than one original report. It also clearly indicates the target performance of a newly developed model for predicting drug-disease associations.

### 2.5 Dataset density and performance

The density of a dataset may be related to the hardness for a method to reach higher performance. We investigate whether there is a relationship between the density of a dataset and the average level of predictive performance that all methods can reach. We still take the most commonly used performance measure AUROC. We average all performance values on a given dataset regardless to the source. We take the most commonly applied dataset, B-Dataset, F-Dataset, C-Dataset and LRSSL (DDA17-763-681) for analysis. The other datasets were not used, due to the number of reported performances on these datasets are not as many as these four. As in Table 2, we can see that, in general, when the performance increase along with the density. Since density is the proportion of known drug-disease associations in all drug-disease pairs, this observation indicates that better prediction results may be easier to obtain on a dataset with higher density of drug-disease association.

**TABLE 2:** Relationship between dataset density and meta performance.

Dataset	Density	Average AUROC
B-dataset	0.115	0.846
F-dataset	0.010	0.865
C-dataset	0.009	0.828
LRSSL	0.006	0.818

The only exception is on the F-Dataset. It appears that all methods have exceptionally high performance on the F-Dataset. The F-Dataset is the earliest benchmarking dataset for predicting drug-disease associations. Most methods applied this dataset as the foundation in their development. Therefore, most methods tend to optimize their results on the F-Dataset.

## 3 Materials and methods

### 3.1 Benchmarking datasets collection

The benchmarking datasets in DDA-Bench were all collected from their original reports. Most of the datasets are collected from GitHub repositories. The exceptions are the DrugNet dataset, the SND dataset (DDA21-867-803) (Jarada et al., 2021), and the TL-HGBI dataset (DDA14-549-233). The DrugNet dataset was retrieved from the authors’ website. The SND dataset was retrieved by using the Wayback machine, due to the missing of the original web page in the paper. The TL-HGBI dataset was downloaded from Google Drive, where the authors shared their datasets. All datasets in DDA-Bench are downloaded along with all annotation resources and the original source codes of each method, if they are provided by the original authors. No additional processing was carried out. This is to ensure that users of DDA-Bench can obtain unmodified resources.

The parameters of a dataset, which includes the number of drugs, the number of diseases, the number of drug-disease associations, and their primary sources are extracted from the original report of each method. We define a dataset density parameter (*ρ*) for each dataset, as follows Equation 1:

$$\rho =\frac{n}{gd},$$ (1)

where *n* is the number of drug-disease associations, g the number of drugs, and d the number of diseases. The dataset density represents the proportion of known drug-disease associations in all drug-disease pairs in a given dataset.

All benchmarking datasets are stored as data packages, which are permanently deposited in the Zenodo archive (https://zenodo.org/records/14719505). DDA-Bench database also keeps local store of all packages, in case users have difficulties in accessing Zenodo. Hyperlinks to the original repositories, as well as the PubMed ID and DOI numbers, are also provided on the page for each dataset. A comprehensive list of all datasets, with annotations for each of them, is provided as Supplementary Table S1. The curation process is illustrated in Supplementary Figure S1.

### 3.2 Performance values curation

We collect performance values from studies in the last 5 years. This is why some important work was included in the benchmarking dataset collection, but not in the performance value collection. The study that enters our performance value collection satisfy the following four conditions. First, it has to be a formally published work in the last 5 years or providing a commonly used benchmarking dataset. Second, it has to be indexed by PubMed or Web of Science. Third, it must include a comparison with state-of-the-art methods when it is published. Fourth, it must provide publicly available full text reports.

We selected 12 studies (Supplementary Table S2) that satisfy all these conditions. The full text of each study is processed by AI-based assistants. Three AI-based assistants are utilized in extracting performance values from academic papers. They are the Kimi K2 chatbot, the Qwen 3.5 chatbot, and the ChatGPT 3.5 chatbot. We use the online free edition of all three AI-assistants. All three AI-assistants are given same prompts along with PDF format full text paper for analysis. The outputs of each AI-based assistant are collected. Formats of the output are manually adjusted. All outputs of AI-based assistant are manually reviewed to ensure their correctness. Manually reviewed and formatted data tables were imported to a MySQL database after removing duplicate records. Prompts for AI-based assistant are provided as in Supplementary Materials.

The performance values are recorded with the reporting study, the method name, the benchmarking dataset, the protocol to obtain the value, the performance measure name and the performance value itself (Supplementary Table S3). The reporting study is the study where the value is obtained. This may not be the study reporting the method itself. It may be another study reporting a comparison result. We collect these results to represent inevitable variations in performance values when comparisons were performed. The protocol to obtain the value is usually one of the ‘5-fold cross-validation’, ‘10-fold cross-validation’, or ‘independent test’. The most common performance measure is the AUROC (Area Under the Receiver Operating Curve). Other performance measures include Precision, Recall, Sensitivity, Specificity, Accuracy, F1-Score, MCC (Mathew’s correlation coefficient), and AUPR (Area Under Precision-Recall Curve). All performance values are obtained directly from literatures, without any modification. The entire data curation processes is illustrated in Supplementary Figure S1B.

### 3.3 Website implementation

We implement the DDA-Bench as an online database service. The DDA-Bench service is written using typescript. The frontend of the DDA-Bench is implemented with Vue. js framework. The backend is built with express. js framework on Node. js. Data tables are saved in a MySQL database. The entire service is hosted on a Debian server. The web service is exposed to internet by a Nginx reverse proxy.

The DDA-Bench service contains five pages. The ‘Home’ page provides an introduction to the entire site, with links to every other pages. The ‘List’ page list all benchmarking dataset in the DDA-Bench database. By clicking dataset name on the left side-bar, details of the database will be shown on the right side, with links to the source package and necessary citation information of the dataset. The ‘Bench’ page list the performance values in the DDA-Bench along with the source information. These values can be filtered by the study where it is reported, by the method it evaluated, or by the benchmarking dataset it applied. The filtered results can be downloaded as a CSV (Comma Separated Vector) table. The ‘Download’ page provides download links to the summary table of DDA-Bench and a comprehensive package containing all benchmarking datasets. The ‘About’ page describes the usage of DDA-Bench briefly.

## 4 Conclusion

By curating benchmarking datasets and performance values in various studies on predicting drug-disease associations, we created the DDA-Bench online database service. The DDA-Bench database facilitates studies on predicting drug-disease associations by providing a unified repository for data resources and baseline performance values. We also provide a comprehensive package that contains all commonly used benchmarking datasets, which saves time and efforts in collecting these resources. The future work of DDA-Bench should be constructing a universal standard benchmarking dataset, which will be the basis for comparing newly developed methods, as well as other state-of-the-art methods.

## Data Availability

Publicly available datasets were analyzed in this study. This data can be found here: https://dda.csbios.net.

## References

[B1] Begley C. G. Ashton M. Baell J. Bettess M. Brown M. P. Carter B. (2021). Drug repurposing: misconceptions, challenges, and opportunities for academic researchers. Sci. Transl. Med. 13, eabd5524. 10.1126/scitranslmed.abd5524 34550729

[B2] Corsello S. M. Bittker J. A. Liu Z. Gould J. McCarren P. Hirschman J. E. (2017). The drug repurposing hub: a next-generation drug library and information resource. Nat. Med. 23, 405–408. 10.1038/nm.4306 28388612 PMC5568558

[B3] Davis A. P. Wiegers T. C. Johnson R. J. Sciaky D. Wiegers J. Mattingly C. J. (2023). Comparative toxicogenomics database (CTD): update 2023. Nucleic Acids Res. 51, D1257–D1262. 10.1093/nar/gkac833 36169237 PMC9825590

[B4] Gottlieb A. Stein G. Y. Ruppin E. Sharan R. (2011). PREDICT: a method for inferring novel drug indications with application to personalized medicine. Mol. Syst. Biol. 7, 496. 10.1038/msb.2011.26 21654673 PMC3159979

[B5] Graham F. (2021). Daily briefing: pfizer’s COVID pill looks promising. Nature. 10.1038/d41586-021-03379-5 34754102

[B6] Gu Y. Zheng S. Yin Q. Jiang R. Li J. (2022). REDDA: integrating multiple biological relations to heterogeneous graph neural network for drug-disease association prediction. Comput. Biol. Med. 150, 106127. 10.1016/j.compbiomed.2022.106127 36182762

[B7] Huang F. Qiu Y. Li Q. Liu S. Ni F. (2020). Predicting drug-disease associations *via* multi-task learning based on collective matrix factorization. Front. Bioeng. Biotechnol. 8, 218. 10.3389/fbioe.2020.00218 32373595 PMC7179666

[B8] Hussain M. S. Mir P. A. Kumar N. Mohi-Ud-Din R. Wali A. F. Mir R. H. (2025). From lab to clinic: success stories of repurposed drugs in treating major diseases. Adv. Pharmacol. Pharm. Sci. 2025, 1070716. 10.1155/adpp/1070716 41116841 PMC12535746

[B9] Jarada T. N. Rokne J. G. Alhajj R. (2021). SNF-NN: computational method to predict drug-disease interactions using similarity network fusion and neural networks. BMC Bioinforma. 22, 28. 10.1186/s12859-020-03950-3 33482713 PMC7821180

[B10] Jiang Z. Li P. (2024). DeepDR: a deep learning library for drug response prediction. Bioinformatics 40, btae688. 10.1093/bioinformatics/btae688 39558584 PMC11629690

[B11] Kim J. H. Scialli A. R. (2011). Thalidomide: the tragedy of birth defects and the effective treatment of disease. Toxicol. Sci. 122, 1–6. 10.1093/toxsci/kfr088 21507989

[B12] Law V. Knox C. Djoumbou Y. Jewison T. Guo A. C. Liu Y. (2014). DrugBank 4.0: shedding new light on drug metabolism. Nucleic Acids Res. 42, D1091–D1097. 10.1093/nar/gkt1068 24203711 PMC3965102

[B13] Li D. Xiao Z. Sun H. Jiang X. Zhao W. Shen X. (2024). Prediction of drug-disease associations based on multi-kernel deep learning method in heterogeneous graph embedding. IEEE/ACM Trans. Comput. Biol. Bioinform. 21, 120–128. 10.1109/TCBB.2023.3339189 38051617

[B14] Luo H. Wang J. Li M. Luo J. Peng X. Wu F.-X. (2016). Drug repositioning based on comprehensive similarity measures and Bi-Random walk algorithm. Bioinformatics 32, 2664–2671. 10.1093/bioinformatics/btw228 27153662

[B15] Luo H. Wang J. Li M. Luo J. Ni P. Zhao K. (2019). Computational drug repositioning with random walk on a heterogeneous network. IEEE/ACM Trans. Comput. Biol. Bioinform. 16, 1890–1900. 10.1109/TCBB.2018.2832078 29994051

[B16] Martínez V. Navarro C. Cano C. Fajardo W. Blanco A. (2015). DrugNet: network-based drug-disease prioritization by integrating heterogeneous data. Artif. Intell. Med. 63, 41–49. 10.1016/j.artmed.2014.11.003 25704113

[B17] Mohan M. Rajendiran K. Rajaram Y. Balaji S. Cassinadane A. (2024). Current trends in pharmaceutical industry: post -CoVid-19 pandemic effects. Bioinformation 20, 1784–1788. 10.6026/9732063002001784 40230918 PMC11993364

[B18] Mohanty S. Harun Ai Rashid M. Mridul M. Mohanty C. Swayamsiddha S. (2020). Application of artificial intelligence in COVID-19 drug repurposing. Diabetes Metab. Syndr. 14, 1027–1031. 10.1016/j.dsx.2020.06.068 32634717 PMC7332938

[B19] Saranraj K. Kiran P. U. (2025). Drug repurposing: clinical practices and regulatory pathways. Perspect. Clin. Res. 16, 61–68. 10.4103/picr.picr_70_24 40322475 PMC12048090

[B20] Su Y.-Y. Huang H.-C. Lin Y.-T. Chuang Y.-F. Sheu S.-Y. Lin C.-C. (2025). HEDDI-Net: heterogeneous network embedding for drug-disease association prediction and drug repurposing, with application to Alzheimer’s disease. J. Transl. Med. 23, 57. 10.1186/s12967-024-05938-6 39891114 PMC11786366

[B21] Suchonwanit P. Thammarucha S. Leerunyakul K. (2019). Minoxidil and its use in hair disorders: a review. Drug Des. Devel Ther. 13, 2777–2786. 10.2147/DDDT.S214907 31496654 PMC6691938

[B22] Wang W. Yang S. Zhang X. Li J. (2014). Drug repositioning by integrating target information through a heterogeneous network model. Bioinformatics 30, 2923–2930. 10.1093/bioinformatics/btu403 24974205 PMC4184255

[B23] Witek T. J. (2021). How the global COVID-19 pandemic brought drug and vaccine development into the public mainstream. Pharm. Med. 35, 287–295. 10.1007/s40290-021-00402-y 34580837 PMC8475305

[B24] Wu G. Liu J. Wang C. (2017). Predicting drug-disease interactions by semi-supervised graph cut algorithm and three-layer data integration. BMC Med. Genomics 10, 79. 10.1186/s12920-017-0311-0 29297383 PMC5751445

[B25] Xuan P. Cao Y. Zhang T. Wang X. Pan S. Shen T. (2019). Drug repositioning through integration of prior knowledge and projections of drugs and diseases. Bioinformatics 35, 4108–4119. 10.1093/bioinformatics/btz182 30865257

[B26] Yang M. Luo H. Li Y. Wang J. (2019). Drug repositioning based on bounded nuclear norm regularization. Bioinformatics 35, i455–i463. 10.1093/bioinformatics/btz331 31510658 PMC6612853

[B27] Yang H. Ding Y. Tang J. Guo F. (2021a). Drug–disease associations prediction *via* multiple kernel-based dual graph regularized least squares. Appl. Soft Comput. 112, 107811. 10.1016/j.asoc.2021.107811

[B28] Yang M. Huang L. Xu Y. Lu C. Wang J. (2021b). Heterogeneous graph inference with matrix completion for computational drug repositioning. Bioinformatics 36, 5456–5464. 10.1093/bioinformatics/btaa1024 33331887

[B29] Yu Z. Huang F. Zhao X. Xiao W. Zhang W. (2021). Predicting drug-disease associations through layer attention graph convolutional network. Brief. Bioinform. 22, bbaa243. 10.1093/bib/bbaa243 33078832

[B30] Zhang W. Yue X. Lin W. Wu W. Liu R. Huang F. (2018). Predicting drug-disease associations by using similarity constrained matrix factorization. BMC Bioinforma. 19, 233. 10.1186/s12859-018-2220-4 29914348 PMC6006580

[B31] Zhang W. Xu H. Li X. Gao Q. Wang L. (2020). DRIMC: an improved drug repositioning approach using Bayesian inductive matrix completion. Bioinformatics 36, 2839–2847. 10.1093/bioinformatics/btaa062 31999326

[B32] Zhao B.-W. Hu L. You Z.-H. Wang L. Su X.-R. (2022). HINGRL: predicting drug-disease associations with graph representation learning on heterogeneous information networks. Brief. Bioinform. 23, bbab515. 10.1093/bib/bbab515 34891172

[B33] Zhong J. Cui P. Zhu Y. Xiao Q. Qu Z. (2023). DAHNGC: a graph convolution model for drug-disease association prediction by using heterogeneous network. J. Comput. Biol. 30, 1019–1033. 10.1089/cmb.2023.0135 37702623

[B34] Zhou Y. Wang F. Tang J. Nussinov R. Cheng F. (2020). Artificial intelligence in COVID-19 drug repurposing. Lancet Digit. Health 2, e667–e676. 10.1016/S2589-7500(20)30192-8 32984792 PMC7500917

