# Supplementary material for: DDA-bench: a manually curated database for benchmarking datasets and baseline performance values in predicting drug-disease associations
Source: Front Genet. 2026 Jan 7;16:1755600. doi: 10.3389/fgene.2025.1755600 (PMC12818788; doi:10.3389/fgene.2025.1755600)
Supplement: Supplementary file 1 [file Presentation1.pptx]

## Slide 1
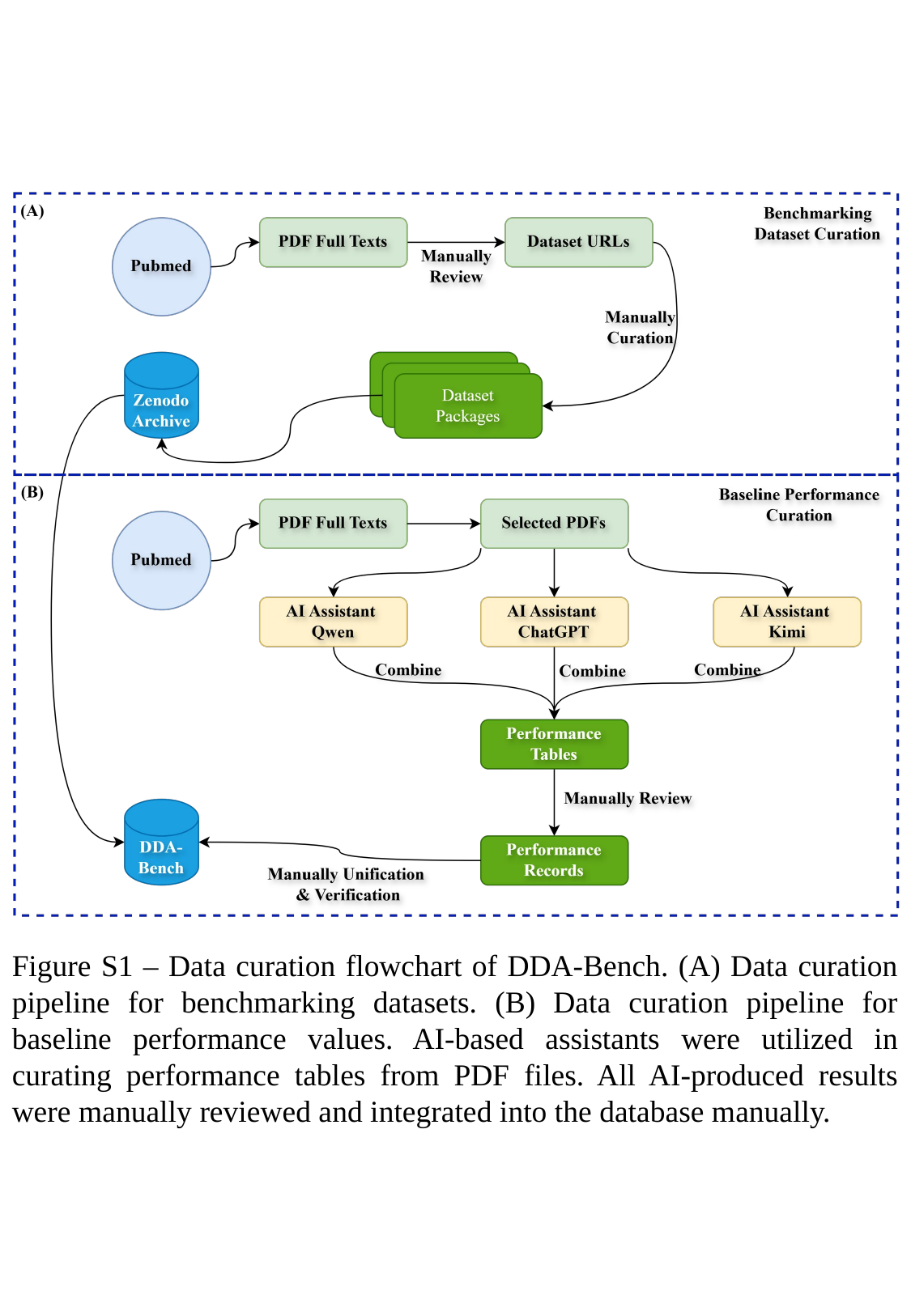

Figure S1 – Data curation flowchart of DDA-Bench. (A) Data curation pipeline for benchmarking datasets. (B) Data curation pipeline for baseline performance values. AI-based assistants were utilized in curating performance tables from PDF files. All AI-produced results were manually reviewed and integrated into the database manually.
