# Supplementary material for: DDA-bench: a manually curated database for benchmarking datasets and baseline performance values in predicting drug-disease associations
Source: Front Genet. 2026 Jan 7;16:1755600. doi: 10.3389/fgene.2025.1755600 (PMC12818788; doi:10.3389/fgene.2025.1755600)
Supplement: Supplementary file 2 [file Table2.docx]

Prompts For ChatGPT

read the open accessed paper from "https://www.frontiersin.org/journals/bioengineering-and-biotechnology/articles/10.3389/fbioe.2020.00218/full ", find out all performance comparisons between CMFMTL and other methods, report all performance comparison as a table

read the CMFMTL paper, find out all performance comparisons between CMFMTL and other methods, report all performance comparison as a table.

convert this table to a format like: Reported_By,Dataset,Method,ValidationProtocol,MetricName,MetricValue. The value of Reported_By field is alwasy "CMFMTD" for all rows.

Read these two papers. Find out all performance comparisons for DD-HGNN and other methods, as well as HMLKGAT and other methods. Report these comparisons as a table. The table includes the following columns: Reported_By,Dataset,Method,ValidationProtocol,MetricName,MetricValue. The "Reported_by" column indicate from which paper this row is collected.

Prompts for Kimi

Read this paper. Find out all performance comparisons between MKDGRLS and other methods. Report all performance comparisons in a table, with this format: Reported_By,Dataset,Method,ValidationProtocol,MetricName,MetricValue. The value for "Reported_by" is alway MKDGRLS.

Ignore Table 11 and all tables later

convert this table to a CSV file for downloading

Read this paper. Find out all performance comparisons between HMLKGAT and other methods. Report all performance comparisons in a table, with this format: Reported_By,Dataset,Method,ValidationProtocol,MetricName,MetricValue. The value for "Reported_by" is alway HMLKGAT.

Convert this table to a CSV file for downloading

Read this paper, find out all performance comprisons between SNF-NN and other methods. report all performance comparisons as a table.

convert all above tables to a format like this: Dataset,Method,ValidationProtocol,MetricName,MetricValue.

add a column as the first column, name the column "Reported_By". All rows for this column have value "SNF-NN", generate a CSV file for downloading

Read this paper, find out all performance comprisons between BNNR and other methods. report all performance comparisons as a table.

Replace the name "Gold Standard" as "F-Dataset", "Cdataset" as "C-Dataset", "DNdataset" as "DrugNet", "De novo (new drugs)" as "Independent test", remove Top 1 or 5 retrieved measure

name metric "AUC (ROC)" as "AUROC", "AUC (PR)" as "AUPR", "Precision (PR)" as "Precision"

convert this table to a format like this: Dataset,Method,ValidationProtocol,MetricName,MetricValue.

as a column as the first column, name the column "Reported_By". All rows for this column have value "BNNR"

generate a csv file for downloading

read this paper and report as a table for all performance comparisons (MRDDA)

For table 2 and 3, convert these tables to a format like this: Dataset,Method,ValidationProtocol,MetricName,MetricValue. Merge these tables as one.

name Bdataset as "B-Dataset", Cdataset and Kdataset similar. remove std part from values.

generate a CSV file for downloading

Prompts for Qwen

read this paper and report as a table for all performance comparisons

convert the above table to a format like Dataset,Method,MetricName,MetricValue

Remove the "Dataset" string from all Dataset names

what kind of validation protocol was used in the paper for comparisons

read this paper and report as a table for all performance comparisons

Convert above table to this format Dataset,Method,ValidationProtocol, MetricName, MetricValue. In this format, name the "Proposed Benchmark" as "K-Dataset"

read this paper. find out all performance comparisons between LRSSL and other methods, report all performance comparisons as a table

name "New Drug Indication Prediction" as "5-fold CV", "Drug Repositioning" as "10-fold CV", "All Drugs" a "LRSSL" , "MAP" as "Precision", remove all items for "Drugs with side effect info"

remove metric HLU and Precision@top-20, convert this to a format like: Reported_By,Dataset,Method,ValidationProtocol,MetricName,MetricValue. The value of Reported_By field is alwasy "LRSSL" for all rows.

read the paper TL-HGBI and its supplemenary document. Find out all performance comparisons between TL-HGBI and other methods, report all performance comparisons as a table

read this paper about method MTRD, find out all performance comparisons between MTRD and other methods. Report all performance comparisons as a table.

keep only AUC and AUPR. name "AUC" as "AUROC", convert this table to a format like: Reported_By,Dataset,Method,ValidationProtocol,MetricName,MetricValue. The value of Reported_By field is alwasy "MTRD" for all rows. remove the "(proposed)".

read the open accessed paper from "https://www.frontiersin.org/journals/bioengineering-and-biotechnology/articles/10.3389/fbioe.2020.00218/full ", find out all performance comparisons between CMFMTL and other methods, report all performance comparison as a table

Read this paper. Find out all performance comparisons between DD-HGNN and other methods. Report all performance comparisons in a table, with this format: Reported_By,Dataset,Method,ValidationProtocol,MetricName,MetricValue. The value for "Reported_by" is alway DD-HGNN.

Read this paper. Find out all performance comparisons between HMLKGAT and other methods. Report all performance comparisons in a table, with this format: Reported_By,Dataset,Method,ValidationProtocol,MetricName,MetricValue. The value for "Reported_by" is alway HMLKGAT.
